# Unique Features of Two Potassium Channels, OsKAT2 and OsKAT3, Expressed in Rice Guard Cells

**DOI:** 10.1371/journal.pone.0072541

**Published:** 2013-08-13

**Authors:** Hyunsik Hwang, Jinyoung Yoon, Hyun Yeong Kim, Myung Ki Min, Jin-Ae Kim, Eun-Hye Choi, Wenzhi Lan, Young-Min Bae, Sheng Luan, Hana Cho, Beom-Gi Kim

**Affiliations:** 1 Department of Molecular Breeding, National Academy of Agricultural Science, Rural Development Administration, Suwon, Korea; 2 NJU-NJFU Joint Institute for Plant Molecular Biology, School of Life Sciences, Nanjing University, Nanjing, China; 3 Departments of Physiology, Konkuk University School of Medicine, Choongju, Korea; 4 Department of Plant and Microbial Biology, University of California, Berkeley, Berkeley, California, United States of America; 5 Department of Physiology, Sungkyunkwan University School of Medicine, Suwon, Korea; Xuzhou Medical college, China

## Abstract

Potassium is the most abundant cation and a myriad of transporters regulate K^+^ homeostasis in plant. Potassium plays a role as a major osmolyte to regulate stomatal movements that control water utility of land plants. Here we report the characterization of two inward rectifying shaker-like potassium channels, OsKAT2 and OsKAT3, expressed in guard cell of rice plants. While OsKAT2 showed typical potassium channel activity, like that of *Arabidopsis* KAT1, OsKAT3 did not despite high sequence similarity between the two channel proteins. Interestingly, the two potassium channels physically interacted with each other and such interaction negatively regulated the OsKAT2 channel activity in CHO cell system. Furthermore, deletion of the C-terminal domain recovered the channel activity of OsKAT3, suggesting that the C-terminal region was regulatory domain that inhibited channel activity. Two homologous channels with antagonistic interaction has not been previously reported and presents new information for potassium channel regulation in plants, especially in stomatal regulation.

## Introduction

Potassium is an essential cation that is indispensable for diverse physiological processes such as turgor adjustment, cell elongation, stomata and leaf movement, generation of the electrochemical membrane potential, and regulation of enzyme activity [1,2]. Stomata movement in particular is directly dependent on changes in osmotic potential that result from K^+^ fluxes in guard cells. As stomata pores are responsible for water loss in leaves, stomatal regulation is considered to be a promising target for developing drought-tolerant crops.

Over the past decades, a great deal of effort has been focused on identifying the molecular mechanisms and signal transduction pathways for stomatal movement in *Arabidopsis thaliana*. It is now known that shaker-like potassium channels are the major players regulating K^+^ transport in guard cells. These K^+^-selective channels typically consist of six trans-membrane domains, a cyclic nucleotide binding domain, and some have an ankyrin repeat domain, and have been characterized in both animals and plants [3,4].

In *Arabidopsis* , there are nine shaker-like potassium channels, which can be divided into three major groups (inward rectifiers, outward rectifiers, weakly inward rectifiers) in terms of their functions, structures, and localizations [5]. *AtAKT1* and *AtKAT1* were the first plant shaker-like potassium channel genes discovered. The *AtKAT1* gene is expressed specifically in guard cells [6–8] and encodes a channel that displays characteristics of typical inwardly rectifying K^+^ channels [6,7,9,10]. Therefore, AtKAT1 plays a role in K^+^ uptake of guard cells when stomata open [11–13]. AtKAT2 was identified as a channel most closely related to AtKAT1 [14], and also found to be expressed in guard cells. However, unlike *AtKAT1*, *AtKAT2* transcripts were also identified in the phloem parenchyma of leaf tissue. In contrast to these inwardly rectifying potassium channels, outward rectifiers, SKOR and GORK, are associated with K^+^-efflux in vascular tissues and guard cells, respectively [15].

Shaker-like potassium channels have been reported in several higher plants, including potato (SKT1), tomato (LKT1), carrot (DKT1, KDC1), maize (KZM1, KZM2, ZMK1 and ZmK2.1), barley (HvAKT1, HvAKT2) [16–22] and rice (OsAKT1, OsKAT1) [23–25]. Monocots including most of the important crops have different guard cell systems from dicots in terms of their morphology and distribution on the leaf epidermis. However, physiological mechanisms of stomata closing and opening are known to be similar between monocots and dicots. It was also known that shaker-like K^+^ channels are highly conserved between monocots and *Arabidopsis* in their amino acid sequences and protein domains. It is expected that the function of shaker-like potassium channels is also conserved in guard cells of both monocots and dicots. Thus, these channels may represent good targets for guard cell engineering to improve drought stress tolerance in crops.

Rice is the largest staple food crop, feeding two third of the population in the world. In addition, rice becomes a model system for monocots with a number of advantages, including a small and completely sequenced genome, an efficient transformation system, and many genetic resources including activation tagging and insertional knockout transgenic lines. We utilize rice as a model to study stomatal control and drought tolerance in crop plants [26]. We hypothesize that KAT-like genes serve as important regulators for stomatal movements in rice. Among the KAT members of rice, OsKAT1 was reported to confer salt stress tolerance in yeast and rice suspension cells, but other KAT genes including OsKAT2 and OsKAT3 have not yet been functionally characterized [24]. Here, we attempted to characterize the OsKAT genes and the channels they encode and unexpectedly found some unique features of these channels that are quite different from what are known on *Arabidopsis* KAT channels.

## Materials and Methods

### Plant materials and growth conditions

Rice (*Oryza sativa* L. ssp. *japonica* cv. Dongjin) was used to isolate genes and to perform the transformations in this study. For sterilization, dehusked rice seeds were treated with 70% alcohol for 3 min., treated with 2% NaClO containing Tween 20 twice for 20 min. each, and then washed eight times in sterilized water. These seeds were germinated and grown in Yoshida nutrient solution, which consists of 1.43 mM NH_4_NO_3_, 0.37 mM NaH_2_PO_4_.2H_2_O, 0.5 mM K_2_SO_4_, 1.0 mM CaCl_2_ and 1.6 mM MgSO_4_.7H_2_O [27]. Seedlings and transgenic rice for gus staining were grown in a controlled chamber for 2 weeks under long day conditions (with a photoperiod of 16 hrs light and 8 hrs dark at 28° C). Leaf sheath, leaf blade, culm, tiller, flower, root and seeds of wild type for total RNA isolation were obtained from mature plants.

### Cloning of three Os*KAT* genes and promoters

The full length cDNAs and promoters of *OsKAT1* (Os01g55200), *OsKAT2* (Os01g11250) and *OsKAT3* (Os02g14840), were amplified from rice cDNA and genomic DNA by PCR. The primers used are listed in Table S1. The PCR products were cloned into the pENTR D-TOPO entry vector (Invitrogen, USA) prior to transfer into destination vectors.

### Construction of transgenic plants and GUS staining

Promoters of *OsKAT1*, *OsKAT2*, and *OsKAT3* in entry vectors were introduced into the plant *GUS* reporter expression vector pBGWSF7 [28]. The resulting promoter fusion GUS reporter constructs and the empty pBGWSF7 vector were transferred into *Agrobacterium tumefaciens* strain LBA4404 by electroporation. Rice transgenic plants were generated by the *Agrobacterium* -mediated co-cultivation method, and transformants were screened based on phosphinothricin (PPT) resistance and subsequently grown in a greenhouse [29]. For GUS assays, T1 plants were grown on 1/2 Murashige and Skoog (MS) medium (supplemented with 1% sucrose and 0.4% phytagel and adjusted to pH5.8) for young seedling staining and on soil for mature plant tissue staining. GUS histochemical staining was performed using the substrate X-Glu [30]. GUS-stained plants and tissues were fixed by washing several times with 70% ethanol until the chlorophyll was completely removed from the tissue.

### RT-PCR and real time quantitative PCR

Total RNA was prepared from two-week-old rice seedlings and from several tissues in mature plants using Trizol reagent (Invitrogen, USA) according to the manufacturer’s instructions. Total RNA was purified further using Qiagen RNeasy columns (Qiagen Inc., USA) and on-column DNase I treatment. Two µg of RNA were reverse transcribed in a 25 µl final reaction volume using oligo dT 14 to 18 primer (Invitrogen Inc., USA) and reverse transcriptase (SuperScript III, Invitrogen Inc., USA). The sequences of the primers used are listed in Table S1. The cDNA (100ng) samples were amplified using 2X Power SYBR Green PCR Master mix (Applied Biosystems, USA) with a MyiQ real time PCR system (Biorad, USA). Data were calculated from C_T_, defined as the PCR threshold cycle number. Relative levels of gene expression were evaluated using the ΔC_T_ method. The ΔC_T_ value was determined by subtracting the endogenous control, *UBIQUITIN 5*, C_T_ value for each sample from the target C_T_ value [31]. Those reactions were repeated three times, and representative results from one experiment were shown.

### Complementation of K^+^ uptake-deficient yeast mutants

*OsKAT1*, *OsKAT2*, and *OsKAT3* full length cDNAs were introduced into the yeast expression vector pYES52-DEST (Invitrogen, USA) and transformed into the K^+^ uptake-deficient yeast strain CY162 (*MATa ade2 ura3 leu2 his3 his4 trk1 delta trk2 delta* :*: pCK64*) [6]. Yeast transformation was performed using LiCl as described by 32. Transformed yeast cells were selected on a minimal solid medium (SDGU medium, which is minimal medium supplemented with amino acids except uracil, 2% galactose, 1% raffinose, clontech Inc., USA) including agar^TM^ bacteriological (Amresco). Complementation tests were performed by spotting serially diluted 5 µl cell suspensions (A_600_ =0.2) on solid media containing different concentrations of KCl, followed by incubation at 28° C for 2-3 days and on SDGU liquid media.

### Heterologous Expression of *OsKAT1*, *OsKAT2*, and *OsKAT3* in CHO cells

CHO cells were cultured in Dulbecco’s modified eagle medium (DMEM with 4,500 mg/l glucose; Gibco, USA) containing 2 mM glutamine, 100U/ml penicillin / streptomycin and 10% FBS (Invitrogen, USA). Cells were transfected with 6 µg of the mammalian expression vector pcDNA6.2 V5-DEST (Invitrogen, USA) containing *OsKAT1*, *OsKAT*2, or *OsKAT3* according to the manufacturer’s protocol (Invitrogen, USA) by using lipofectamine 2000. Transfected cells were incubated in DMEM medium and maintained at 37 °C in a humidified incubator in the presence of 5% CO_2_ (Sanyo, Japan). For electrophysiological studies, cells were spread on cover slips coated with 0.1% poly-D-lysine (Sigma-Aldrich, USA). Experiments were performed using cells incubated for 1-3 days after transfection.

### Patch clamp analysis

Current measurements were made with the whole-cell patch clamp technique. Voltage clamp was performed by using an EPic-8 amplifier (HEKA Instruments, Germany) and low-pass filtered at 2 kHz. The patch pipettes (World Precision Instruments, Inc., USA) were made by a Narishige puller (PP-830, Narishige, Japan). The patch pipettes used had a resistance of 2–3 MΩ, when filled with the pipette solutions. The pipette capacitance was compensated after formation of a gigaohm seal. The voltage-clamp protocol for recording KAT currents consisted of stepping the membrane potential from a holding potential of -20 mV to the test potential (ranging from -140 mV to +60 mV) for 1 s in 20 mV increments at 5 s intervals. To minimize changes in offset potentials during bath solution exchanges, 3M-KCl agar salt bridges were used for the reference electrode. The standard bath solution contained 170 mM K-gluconate, 1 mM CaCl_2_, 2.5 mM MgCl_2_, 10 mM HEPES/Tris pH 6.8. The pipette solution contained 150 mM K-gluconate, 20 mM KCl, 1 mM CaCl_2_, 10 mM HEPES, 10 mM EGTA, 4 mM Mg–ATP/Tris pH 7.4. [K]_o_ were varied while osmolarities were maintained with NaCl. Unless otherwise indicated, chemicals were obtained from Sigma-Aldrich. Data are presented as means ± S.E., and the statistical significance of differences were determined by the Student’s *t* test.

### Yeast two hybridization assay

Yeast two-hybridization assays were performed using the MatchmakerTM GAL4 Two-hybrid system 3 (Clontech Inc., USA). C-terminal region stretched into cytoplasmic region of OsKAT2 and OsKAT3 was cloned into pGADT7 and pGBKT7. Each pairs of constructs were co-transformed into *Saccharomyces cerevisiae* strain AH109 and subsequently plated on SD minimal media (Clontech Inc., USA) without leucine and tryptophan, then transferred to selection media without leucine and tryptophan and histidine supplemented with 1.5mm 3-AT (3-amino-1, 2, 4-triazole). Growth was assessed 4 days after transfer.

### Isolation of rice protoplasts and bimolecular fluorescence complementation test

Protoplasts were isolated from 7 days rice seedlings grown in long day conditions. Grown rice plants were chopped with razor blade into the 2-3 mm strips and then dipped into the enzyme solution (1.5% cellulose R-10 (Yakult, Honsa Co., Ltd, Tokyo, Japan), and 0.75% macerozyme R-10 (Yakult Honsha, Japan), 600 mM mannitol, 3.4 mM CaCl2, 5 mM β-mercaptoethanol, 1% of BSA and 10 mM MES, pH 5.6) with gentle shaking (50-60 rpm) at 28 ^°^C for 4-5 hr. The digested samples passed through 100 µm nylon meshes for discarding undigested plants and then passed protoplasts solution was exchanged with W5 solution (154 mM NaCl, 125 mM CaCl2, 5 mM KCl, and 2 mM MES, adjusted to pH 5.7) with dilution and centrifugation with 100 g [33].

For bimolecular fluorescence complementation assay, we taggedN-terminal and C-terminal fragments of YFP to N-terminal and C-terminal of OsKAT2 and OsKAT3, respectively. Amplified *OsKAT2* and *OsKAT3* using PCR reaction was cloned into pSPYCE(M) and pSPYNE(R) (Waadt et al., 2008). Each constructed plasmid DNAs were introduced into rice protoplasts using the PEG-mediated method [34]. Images of protoplasts were obtained using a Zeiss AxioCam MRc CCD camera and a Zeiss Axioimager M1 fluorescence microscope (Carl Zeiss, Jena, Germany). The filter set, XF116 (exciter, 474AF20; dichroic, 500DRLP; emitter, 510AF23) (Omega, Brattleboro, VT) was used for detection of YFP signals. The data were processed using Adobe Photoshop software (Mountain View, CA) and the images were presented in pseudocolor.

## Results

### Identification of three AtKAT1 orthologues in rice

**Figure 1:**
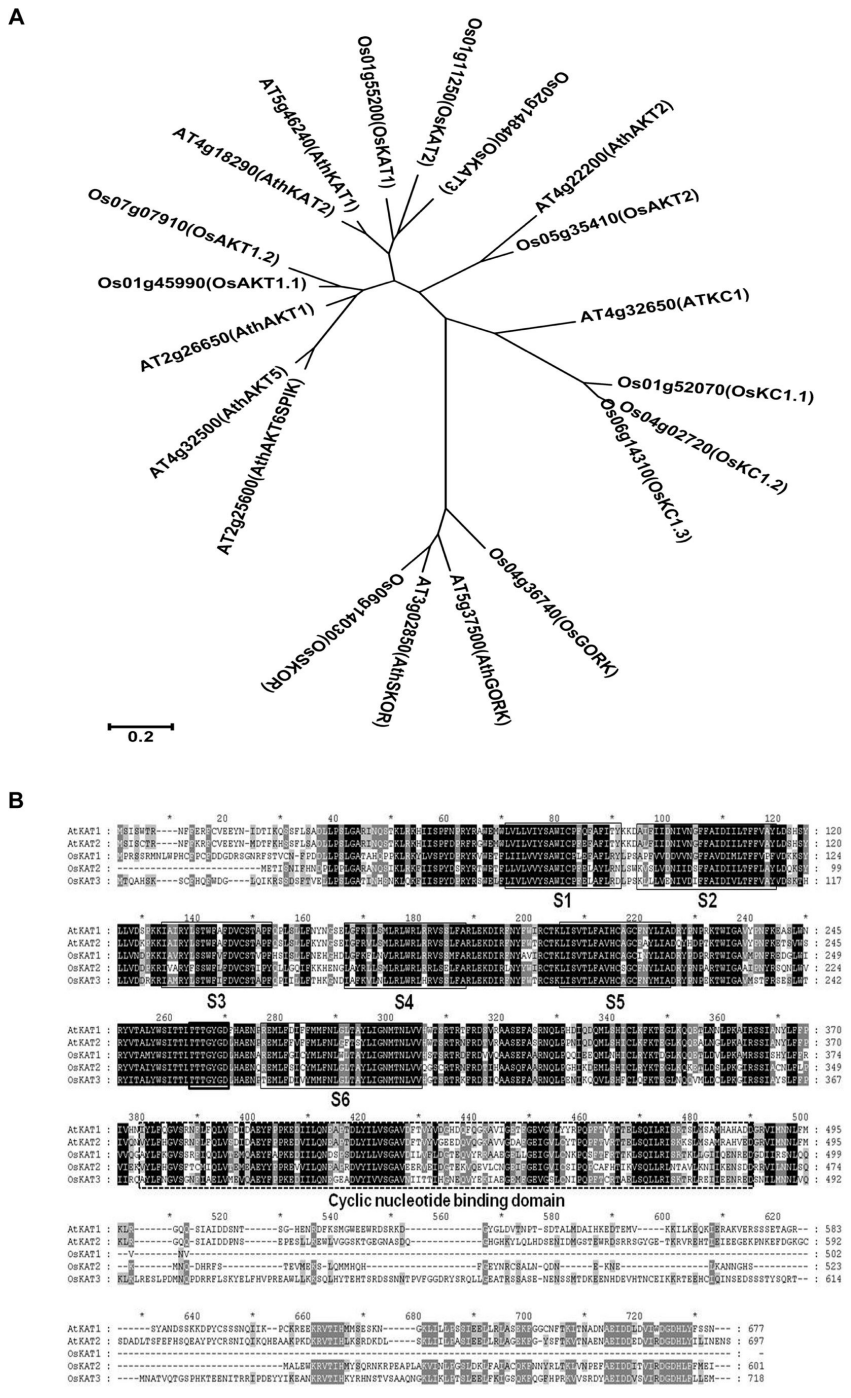
Phylogenetic analysis of plant potassium channels and amino acid sequence alignment of four KAT potassium channel proteins. A, Phylogenetic tree derived from an amino acid sequence alignment of *Arabidopsis* and rice potassium channel proteins. The tree was generated using the Mega 5.0 program by maximum likelihood method. The tree is drawn to scale, with branch lengths measured in the number of substitutions per site. B, The amino acid sequences of three OsKATs from rice, AtKAT1 and 2 from *Arabidopsis* were aligned using the program CLUSTALX. Identical amino acids are shaded. The consensus TXGYGD motif in the K^+^-selective pore-forming loop region is indicated in the box drawn with bold line. The regions in the boxes drawn with solid lines are transmembrane domains (S1-S6).

To identify the KAT subfamily of potassium channel genes in rice, we used amino acid sequence of *AtKAT1* (At5g46240) as the query to perform a BLAST search in the Rice Genome Annotation Project (http://rice.plantbiology.msu.edu/). After using e-value > 2e-15 as a cut-off, the resulting homologous protein sequences were manually checked for the motifs and domains of shaker-like family proteins. As a result, eleven genes were identified to encode shaker-like potassium channels that can be divided into 5 clades in a phylogenetic tree in comparison with the nine *Arabidopsis* shaker-like potassium channels (Figure 1A). Putative rice shaker-like potassium channels consist of two orthologues of AtAKT1, three orthologues of AtKAT1 and AtKAT2, one orthologue of AtAKT2, three orthologues of KC1, and two orthologues of GORK and SKOR. The rice proteins all have the typical domains of shaker-like K^+^ channels, including the conserved transmembrane domains, the GYGD motif of the pore-forming region, and a C-terminal region containing a putative cyclic nucleotide binding domain, and an ankyrin repeat domain in the AKT-type channels.

Amino acid sequence alignment of the three OsKAT proteins with AtKAT1 and AtKAT2 highlights several highly conserved domains (Figure 1B). The most obvious differences in the OsKAT sequences are found in the C-terminal region after the cyclic nucleotide binding domain (CNBD). OsKAT1 does not have the C-terminal region after the CNBD domain at all, and OsKAT3 has longer C-terminal region than that of OsKAT2.

### *OsKAT2* and *OsKAT3* are expressed in guard cells

**Figure 2:**
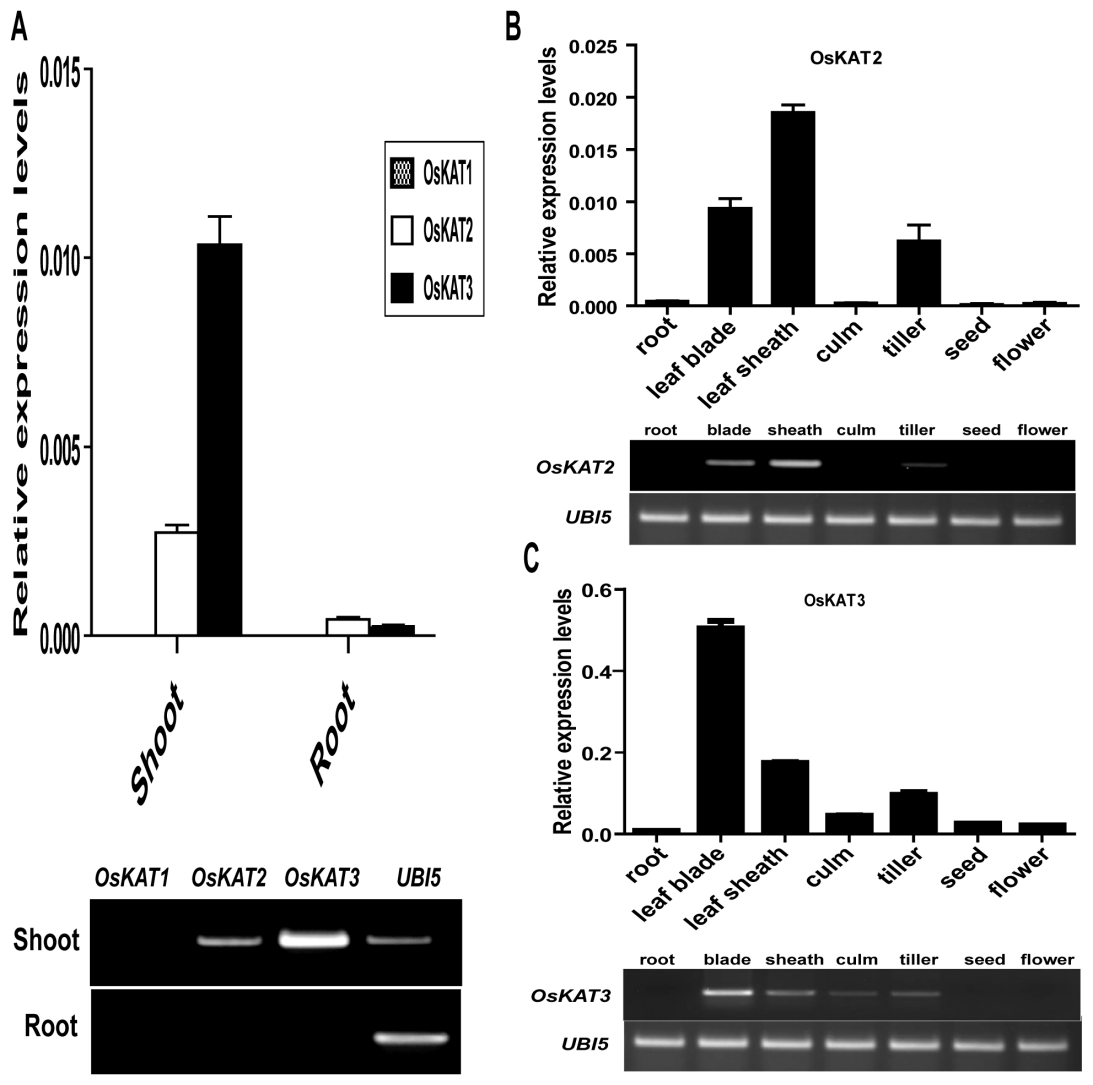
Relative expression levels of three OsKATs genes of Dongjin rice in various tissues. A, Semi-quantitative RT-PCR and Real time PCR were performed for *OsKAT1, OsKAT2, OsKAT3*, and *UBI5* (internal control) using total RNA was isolated from shoots and roots of rice two week oldseedlings. B and C, Semi-quantitative RT-PCR and Real time PCR were performed for *OsKAT2* and *OsKAT3* using the total RNA isolated from roots, leaf blades, leaf sheaths, culm, tillers, flowers and seeds of mature plants.

AtKAT1 functions in K^+^ uptake of guard cells and is expressed specifically in guard cells. In order to elucidate the function of the three *OsKAT* genes, we examined the expression pattern of these genes in rice seedlings. Results from quantitative PCR (qPCR) indicated that Os*KAT1* mRNA was barely detectable in either shoots or roots. Transcripts of *OsKAT2* and *OsKAT3* were detected in shoots but not in roots (Figure 2A). We analyzed expression pattern of *OsKAT2* and *OsKAT3* in more detail using total RNA samples from leaf blade, sheaths, internodes, tillers, flowers, and seeds. High levels of *OsKAT2* and *OsKAT3* transcripts were found in leaf sheaths, leaf blades, and tillers of mature plants (Figure 2B and 2C). Additionally *OsKAT3* was expressed in culm including node and internode (Figure 2C). These data suggest that *OsKAT2* and *OsKAT3* have overlapped expression in aerial parts of the plants.

**Figure 3:**
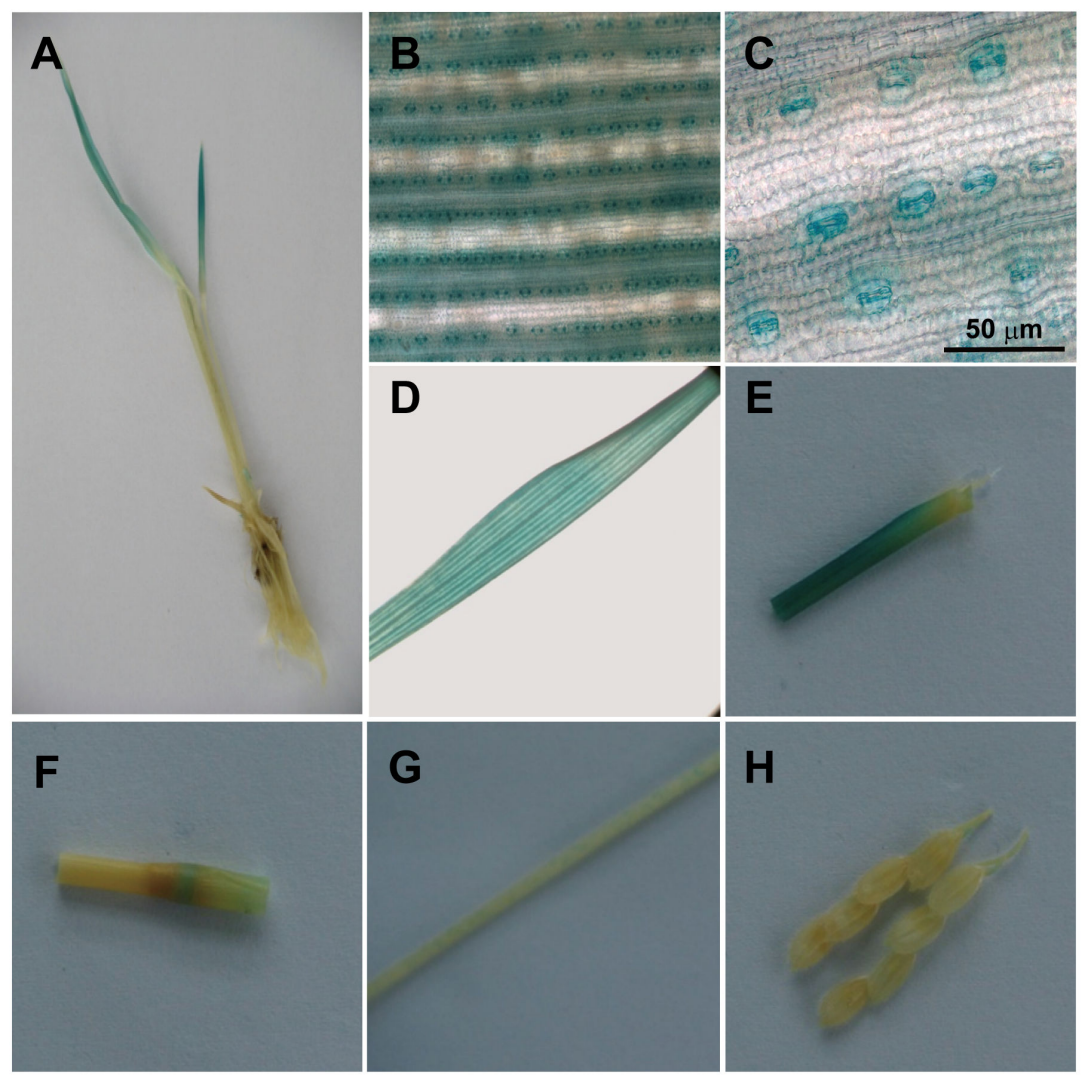
The Os*KAT2* promoters mediate guard cell specific expression of *GUS*. A, A two week old *pOsKAT2::GUS* transgenic seedling. B and C, Guard cells exhibited strong levels of GUS expression in the lower part of the leaf blade. D, E, F, G and H, Leaf blade, leaf sheath, culm, tiller and flower of mature plant were stained by X-glu respectively.

**Figure 4:**
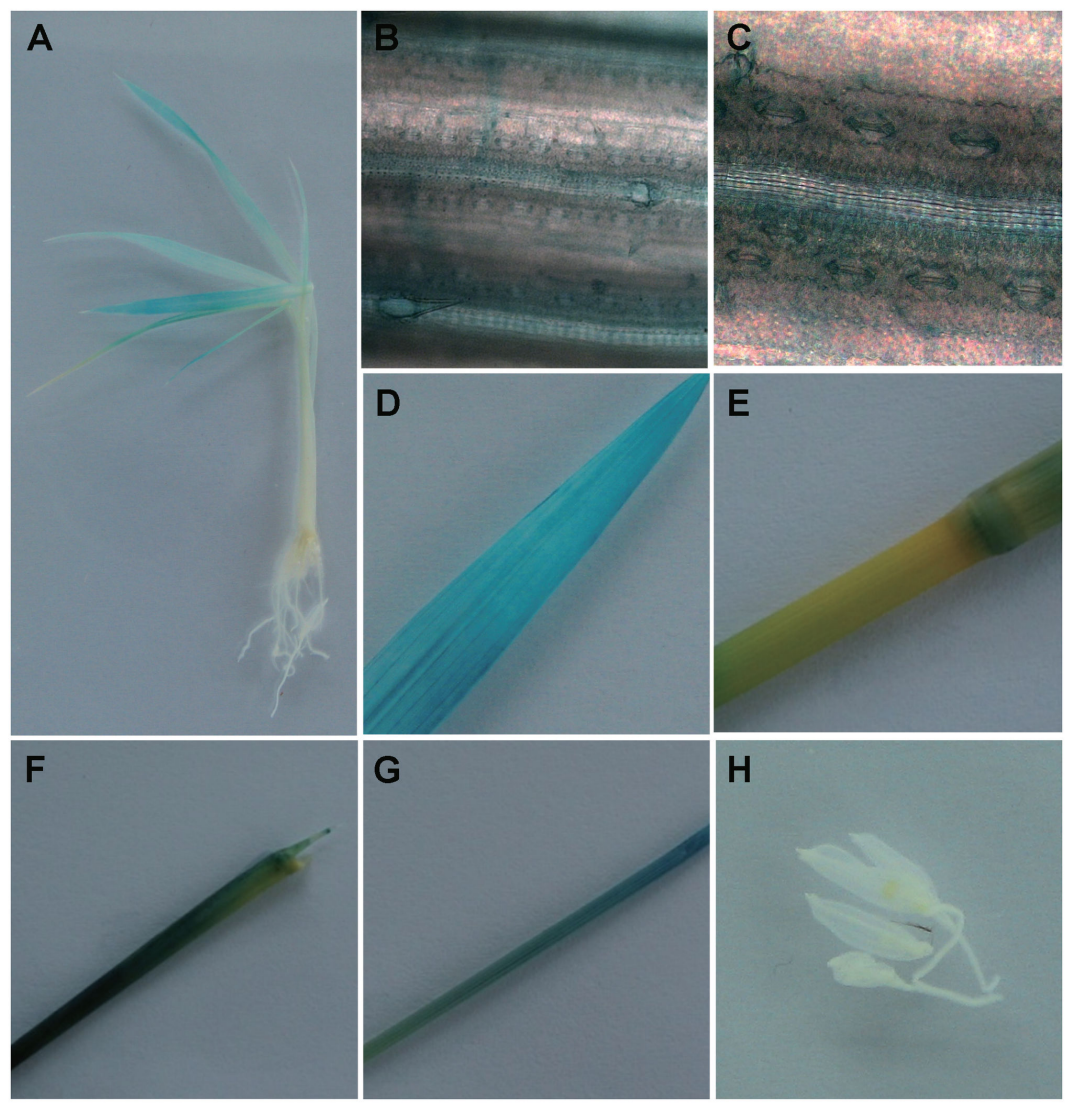
The Os*KAT3* promoters mediate *GUS* expression in aerial tissues including guard cell. A, A two week old *pOsKAT3::GUS* transgenic seedling. B and C, Guard cells exhibited strong levels of GUS expression in the lower part of the leaf blade. D, E, F, G and H, Leaf blade, leaf sheath, culm, tiller and flower of mature plant were stained by X-glu respectively.

We further analyzed the tissue- and cell-specific expression of *OsKAT2* and *OsKAT3* by using transgenic plants harboring the *GUS* reporter gene under the control of the *OsKAT2* and *OsKAT3* promoter regions. Two-week-old seedlings and flowering plants were used to represent young and mature plants, respectively. The activity of the *GUS* reporter gene was examined in five independent T1 transgenic lines by histochemical staining. In young seedlings, strong staining was detected in the shoots but was not in the roots of OsKAT2-GUS and OsKAT3-GUS plants, consistent with the expression patterns by qPCR (Figures 3 and 4). In further microscopic studies, we found clear blue staining specifically localized in the guard cells of *OsKAT2* -*GUS* plants, both young and mature (Figure 3B and C). In the case of *OsKAT3*-GUS plant, blue staining was observed in all the leaf tissues including guard cells, vascular tissues, and mesophylls. In mature plants, we detected GUS activity in different tissues. Expression activities of both *OsKAT2* and *OsKAT3* promoter was strongly detected in limited leaf organs such as leaf blades and sheaths (Figures 3A and E, and 4A and F). However, both of them didn’t show any activity in flowers, root (Figures 3A and H, and 4A and H).

### OsKAT1 and OsKAT2, but not OsKAT3, complement K^+^-uptake-deficient yeast mutant

**Figure 5:**
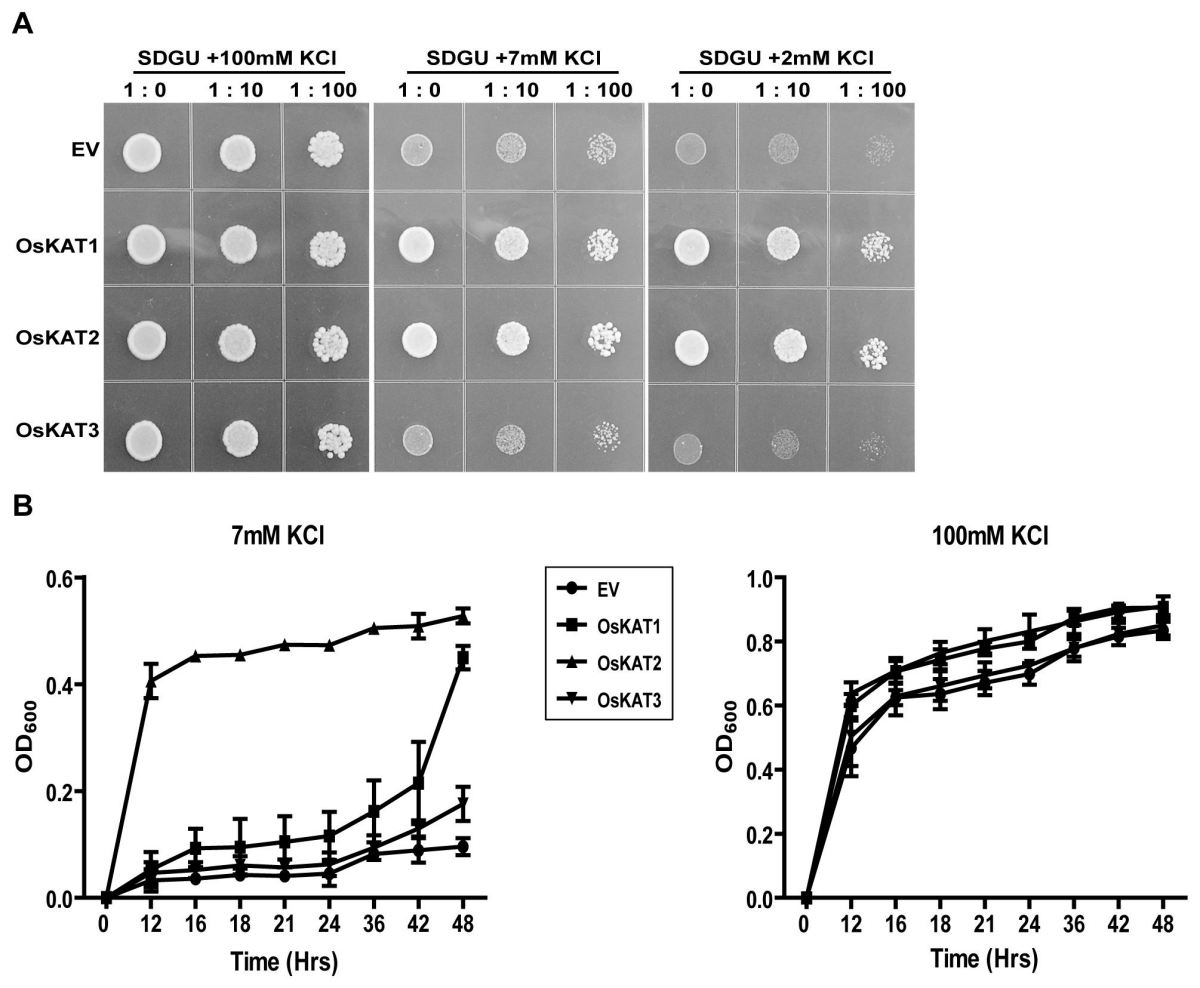
Effect of rice Shaker-like potassium channel expression on growth of a K^+^ uptake-deficient yeast strain. A, CY162 yeast with the empty vector (EV, pYES-DEST52), or expression constructs for rice OsKAT1, OsKAT2, or OsKAT3 were serially diluted (no dilution (1: 0), diluted 1:10 or 1:100 with sterile distilled water) and plated on solid SDGU medium containing 0, 2, 7, or 100mM KCl. B, Time course of cell density (optical density at 600nm; OD_600_) in suspension cultures using liquid SDGU medium containing 7 mM or 100 mM KCl. Data represent means of three cultures grown separately in space and time.

To test whether the three putative OsKAT channels can function in K^+^ uptake, we performed complementation assay using a yeast mutant strain, CY162, that is deficient in the high affinity K^+^ transporters TRK1 and TRK2. CY162 yeast cells fail to grow on media containing K^+^ concentrations lower than -2 mM unless they express exogenous proteins that mediate K^+^ uptake, such as AtKAT1 [6]. When CY162 yeast cells were transformed with the empty pYES52-DEST vector, the cells did not grow well on low K^+^ (<2 mM) media in our experiments. However, yeast cells expressing Os*KAT1* or Os*KAT*2 grew well on agar plate containing 2mM K^+^ (Figure 5A). To our surprise, yeast cells expressing OsKAT3 did not grow well, just like the cells transformed with the empty vector (Figure 5A).

In liquid culture with 7 mM K^+^, CY162 cells containing *OsKAT1* or *OsKAT2* also showed significantly higher growth rates than CY162 cells containing *OsKAT3* or the empty vector. The growth rate with *OsKAT2* was higher than that with Os*KAT1* during the early growth phase. In other words, *OsKAT1-*expressing cells required more time to reach the exponential phase than cells expressing *OsKAT2*. Taken together, of the three putative OsKAT channels, OsKAT1 and OsKAT2, but not OsKAT3, were found to mediate K^+^ influx in yeast cells. This indicates that OsKAT1 and OsKAT2 are likely to be functional inward K^+^-channels in rice. Furthermore, OsKAT2 appears to have a higher capacity for K^+^ uptake than OsKAT1.

### Electrophysiological analysis of OsKAT channels

**Figure 6:**
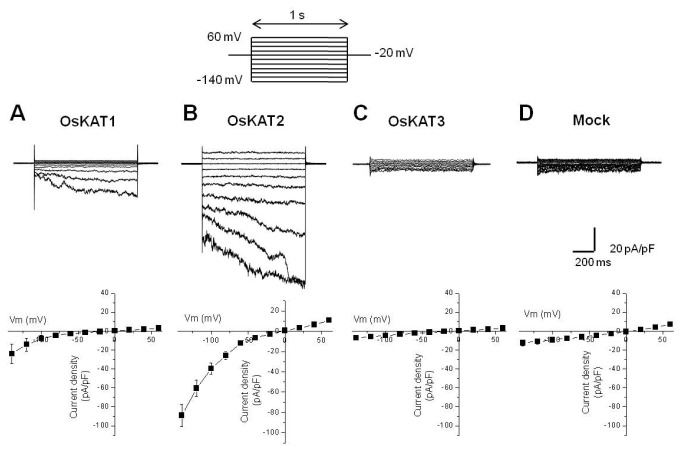
OsKAT1 or OsKAT2 expressed in CHO cells gives rise to voltage- and time-dependent inwardly rectifying K^+^ currents. Current responses of cells in the control bath medium with 170 mM K-gluconate to the standard voltage protocol (inset: -20 mV holding potential, test potentials between -140 and +60 mV in steps of 20 mV) were recorded in the whole cell configuration. *Upper*, representative current traces elicited by command potential in cells transfected with various DNA as indicated. Lower, steady-state I/V relations of current densities collected at the end of test pulses as a function of clamp voltage are shown (n=5, 28, 19, and 13, from A to D, respectively).

**Figure 7:**
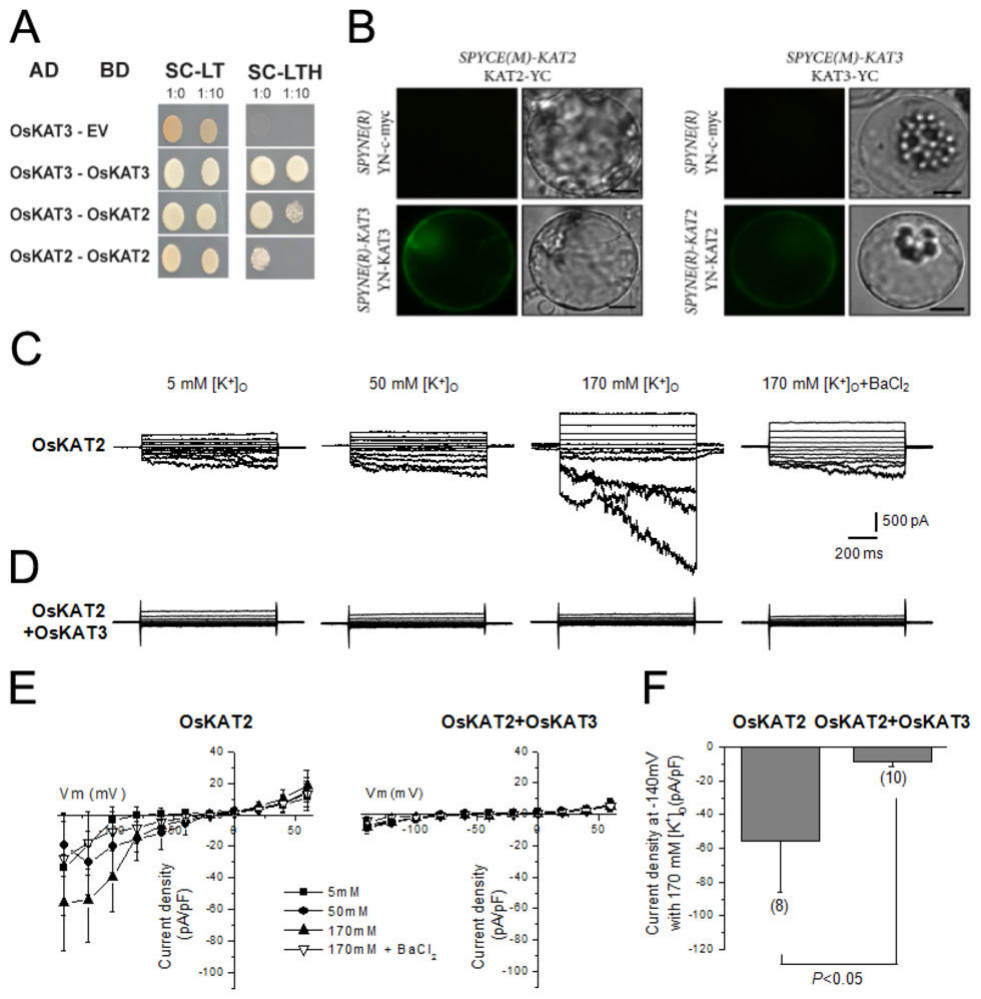
OsKAT2 and OsKAT3 interact each other and OsKAT3 negatively regulate the OsKAT2. A, To test protein interactions between OsKAT2 and OsKAT3, yeast two hybridization was performed using cytosolic c-terminal region of OsKAT2 and OsKAT3. Transformed yeast strains were plated onto SD/-Leu/-Trp/-His medium including 1.5mM 3-AT (right panel) and onto SD/-Leu/-Trp medium as control (left panel). B, *In vivo* interaction test with bimolecular fluorescence complementation (BiFC) assay in rice protoplasts, interactions of YN-c-myc and OsKAT2-YC or OsKAT3-YC (negative control) are presented at upper panels and interactions of YN-OsKAT3 and OsKAT2-YC or YN-OsKAT2 and OsKAT3-YC are showed at lower panels. Bar = 10 µm. C–D, representative whole cell currents recorded in CHO cells expressing OsKAT2 or OsKAT2 with OsKAT3. Currents were obtained by changing the membrane voltage from a holding potential of -20 mV followed by test pulses from -140 mV to +60 mV in 20 mV increments in the presence of various concentrations of external potassium, as indicated. E, steady-state current-voltage curves of OsKAT2 (left) or OsKAT2+OsKAT3 (right) in the presence of 5 mM (■), 50 mM (●), and 170 mM (▲) external potassium. Results are displayed as means ± S.E., with *n* =3, 5, and 8 CHO cells transfected with *OsKAT* or n=10 CHO cells transfected with OsKAT2 +OsKAT3, respectively. Ba^2+^ inhibition of OsKAT2-mediated K^+^_in_ currents (▽) (n=12). E. Current densities of OsKAT2 and OsKAT+OsKAT3 at -140 mV in 170 mM [K^+^]_o_. The numbers of samples used in the analysis are shown in parentheses.

To examine the K^+^-uptake capacity of OsKAT channels, we directly measured the currents of each K^+^ channel expressed in CHO cells. In control experiments, K^+^-current was not observed in mock-transfected CHO cells (Figure 6D). In contrast, large inward currents were observed in OsKAT2-expressing CHO cells (Figure 6B). Voltage pulses negative to 0 mV elicited slowly activating, voltage-dependent inwardly rectifying currents in OsKAT2-expressing cells. OsKAT1 also produced similar inward rectifying currents though the amplitude was smaller (Figure 6A). However, OsKAT3 failed to show channel activities (Figure 6C), consistent with the result in yeast cells where it failed to complement K^+^-uptake deficiency. To compare the channel activities, the current-voltage relationships were obtained from the steady-state currents (Figure 6, bottom). Both OsKAT1 and OsKAT2 showed strong inward rectification but OsKAT2 had a higher current density than OsKAT1 at the same voltage. For instance, at -140 mV, current density of OsKAT1 and OsKAT2 was -24.04±10.27 pA/pF (n=4) and -82.69±10.92 pA/pF (n=11), respectively (p<0.01). Standard solutions contained the impermeable anion gluconate as the counterion for K^+^, thus suggesting that K^+^ is the main carrier of the observed currents elicited by hyperpolarizing pulses. We concluded that OsKAT1 and OsKAT2, but not OsKAT3, function as inwardly rectifying potassium channels. This result is well matched with the results of the yeast growth experiment (Figure 5). To further characterize the OsKAT2 channel, we examined OsKAT2-mediated currents with different external K^+^ concentrations. When the external K^+^ concentration was changed from 5 to 170 mM, the inward current increased by 10.7 fold (Figure 7C), but the shape of current–voltage relationships remained the same (Figure 7E). The *E*rev of the OsKAT2-mediated currents followed approximately the calculated value of the Nernst potential for K^+^ (Figure 7C and E). We also tested sensitivity of OsKAT2 to Ba^2+^, a typical inhibitor of inward K^+^ channels. Application of Ba^2+^ in the bath solution caused a decrease of the inward current (Figure 7C and E). These data verify the notion that OsKAT2 functions as a typical inwardly rectifying K^+^ channel.

### OsKAT3 physically interacts with and inhibits the activity of OsKAT2

The C-terminal cytoplasmic domains of plant K^+^ channels play a role in the assembly of the homo- or hetero-tetrameric channels [35,36]. We hypothesized that the C-terminal regions of OsKAT2 and OsKAT3 may be required for the assembly of the channels. Our results earlier showed that OsKAT2 homo-tetramers are functional channels whereas OsKAT3 homo-tetramers are not. We tested the possibility that OsKAT3 may be functional only after forming hetero-tetramers with other homologous channel proteins such as OsKAT2. We thus investigated whether OsKAT2 and OsKAT3 physically interact with each other through the C-terminal region. Using yeast two hybridization assays, we found that C-terminal regions of OsKAT2 and OsKAT3 can interact with themselves and with each other (Figure 7 A). The homo and hetero combination showed different interaction strengths. The combination of OsKAT3-OsKAT3 showed the strongest interaction and followed by combination of OsKAT2- OsKAT3 and OsKAT2-OsKAT2. This result suggests that OsKAT2 and OsKAT3 can form both homo- and hetero-tetramers via the C-terminal region.

To investigate the interaction between OsKAT2 and OsKAT3 *in vivo*, we performed bimolecular fluorescence complementation assay. *YN-cMyc* and *OsKAT2-YC* or *YN-cMyc* and *OsKAT3-YC* were introduced into rice protoplasts with polyethylene glycol (PEG)-mediated transformation as negative controls. There were no significant signals. In the contrary, when introduced with *YN-OsKAT2* and *OsKAT3-YC* or *YN-OsKAT3* and *OsKAT2-YC* into rice protoplasts, we precisely detected yellow fluorescence signals in plasma membrane area (Figure 7 B). These results are raising the possibility of interaction of OsKAT2 and OsKAT3 *in vivo*.

We tested the possibility that the formation of heterotetramer by OsKAT2 and OsKAT3 may result in changes of channel activity by co-expressing the two in the CHO cell and characterizing the channel activity. To our surprise, CHO cells co-transfected with both channels did not show channel currents under all external K^+^ concentrations (Figure 7 D). The data were summarized in Figure 7 D, E and F. These results suggest that OsKAT3 might negatively regulate OsKAT2 channel activity.

### The C-terminal region of OsKAT3 serves as autoinhibitory domain of its channel activity

**Figure 8:**
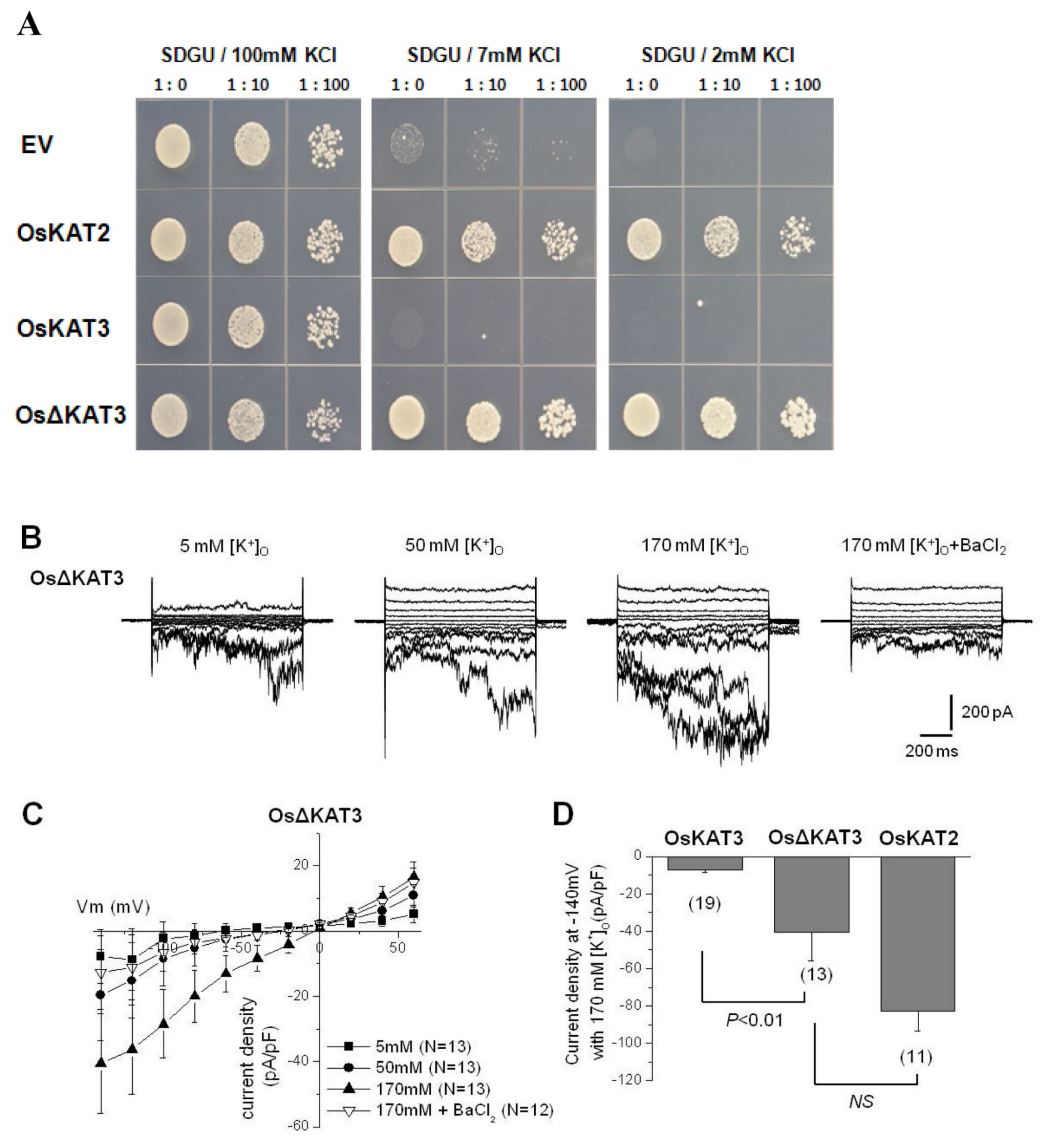
The deletion form of OsKAT3 can recover the inward rectifying K^+^ channel activity in yeast and CHO cell. A, CY162 yeast with the empty vector (EV, pYES-DEST52), or expression constructs for rice OsKAT2, OsKAT3 or deletion form of OsKAT3 (OsΔKAT3) was serially diluted (no dilution (1: 0), diluted 1:10 or 1:100 with sterile distilled water) and plated on solid SDGU medium containing 0, 2, 7, or 100mM KCl. B, representative whole cell currents recorded in CHO cells expressing C-terminal deleted OsKAT3. Currents were obtained by changing the membrane voltage from a holding potential of -20 mV followed by test pulses from -140 mV to +60 mV in 20 mV increments in the presence of various concentrations of external potassium, as indicated. C, steady-state current-voltage curves of whole cell recordings in the presence of 5 mM (■) (n=13), 50 mM (●) (n=13), and 170 mM (▲) (n=13) external potassium, respectively. Ba^2+^ inhibition of OsKAT3-mediated K^+^_in_ currents (▽) (n=12). D, Current densities of OsKAT3, OsKAT3 and OsKAT2 at -140 mV in 170 mM [K^+^]_o_. The numbers of samples used in the analysis are shown in parentheses.

KAT family channels all contain a C-terminal cytosolic region that serve as an interaction domain between channels and other regulatory components [37]. As OsKAT3 inhibits OsKAT2 activity by interacting through the C-terminal regions, we speculate that the same C-terminal region in OsKAT3 may be involved in regulating its own channel activity. Indeed, compared to OsKAT2 and OsKAT1, OsKAT3 has a longer c-terminal region. Thus, to investigate whether this C-terminal region serves as a negative regulatory region, C-terminal region of OsKAT3 behind the nucleotide-binding domain was deleted and the truncated channel was tested for activity in yeast and CHO cells. Yeast complementation assay showed that the OsKAT3 without the C-terminal region complemented the K^+^-uptake deficient yeast mutant CY162 under low K^+^ condition, just as OsKAT2 or OsKAT1 did (Figure 8 A). It suggests that the truncated channel has K^+^-transport activity.

When the same truncated OsKAT3 was expressed in CHO cells, we observed large inward currents activated by hyperpolarization pulses from -60 mV to −140 mV (Figure 8B). When the external K^+^ concentration was increased from 5 to 170 mM, steady-state current amplitude increased and *E*rev followed the calculated value of the Nernst potential for K^+^ (Figure 8B to D). At -140 mV, truncated OsKAT3 elicited -40.59±15.33 pA/pF(n=13), vs. full-length OsKAT3 -7.13±1.27 pA/pF, n=19). The results observed using both yeast and CHO cells suggest that the C-terminal region of OsKAT3 inhibits the channel activity.

## Discussion

Shaker family K^+^channels have been well conserved among eukaryotes. Several shaker-like channels have been identified in higher plants where they play important roles in K^+^ homeostasis. Although these channels are studied extensively in *Arabidopsis* [2], little is understood on the similar channels in rice, the model plant of monocots except for OsKAT1 [24]. We intended to identify the K^+^ channels that control stomatal movements and may provide a target for engineering drought tolerance in crop plants. During the study, we discovered some unique features of rice KAT channels. Particularly interesting point is the negative regulatory function of the C-terminus of OsKAT3 and physical interaction of OsKAT2 and OsKAT3, which leads to silencing of the OsKAT2 channel.

All the KAT channels studied so far display typical inward rectifying currents. The C-termini of these channels mainly function in the interaction among subunits for assembly of tetrameric channels [2,14,35,38]. In one study, the C-terminus of AtKAT1 appears to modulate voltage sensitivity of the channel [36]. In contrast to the general notion, we found that OsKAT3 is silent in CHO cells and, furthermore, deletion of the C-terminal region rendered the channel active. This finding indicates that the C-terminal region, a soluble domain located in the cytosol, serves as an autoinhibitory domain for the channel activity. Autoinhibitory domains have been found in some enzymes such as protein kinases [38]. Some transporters such as CAX also contain such domains that control the transport activity [39]. In all cases, inhibition by such inhibitory domain can be “relieved” in response to intra- or extracellular signals. In the case of protein kinases, calcium elevation in response to various signals activates the kinases through calcium sensors calmodulin or calcineurin B-like proteins [40]. We propose that OsKAT3 may be activated by a signaling pathway that modulates the C-terminal region of the channel protein.

Shaker family channels function as tetramers that can be assembled by four of the same subunits (homotetramers) or four of the different subunits (heterotetramers) [35]. Almost all of them can function as homotetramers as demonstrated by functional analysis after expression of a single gene encoding one channel protein [41]. Our analysis on OsKAT2 is one example: expression of the *OsKAT2* gene alone produces inward rectifying currents in the CHO cells. Examples of hetero-tetramer channels include that formed by AtKAT1 and AtKAT2: these two subunits interact and form both hetero-tetramer or homo-tetramer to function. Both channels can function as homo-tetramers, but preferential AtKAT1-AtKAT2 interaction appears to produce currents that are slightly different from any of the homo-tetramer channels [36]. Strikingly different is the finding in our study on OsKAT2 and OsKAT3: while OsKAT2 alone is active, co-expression of the two channels completely silenced the channel activity of OsKAT2. This dramatic effect caused by interaction among the KAT subunits represents a new mechanism for channel regulation. Such mechanism may be particularly interesting when considering the expression pattern of the two channel genes in rice plants. Previously expression patterns of OsKAT1, 2, and 3 in different tissues were confirmed by Northern analysis. OsKAT1 was only detected in the internode and rachides. OsKAT2 and OsKAT3 were all expressed in different tissues of aerial part of rice even if OsKAT3 were expressed higher than OsKAT2 [24]. These results were similar with our data using Q-PCR and we showed the cell specific expression pattern using promoter-gus analysis. The *OsKAT2* gene is predominantly expressed in guard cells, consistent with the hypothesis that this may be the functional orthologue of *Arabidopsis* KAT1, *OsKAT3* gene has a broader expression profile including guard cells and other leaf tissues. As OsKAT3 and OsKAT2 are both present in the guard cells, we propose that depending on the expression levels of these two proteins, the ratio of OsKAT2 homotetramer and OsKAT2-OsKAT3 heterotetramer will vary and thus control the activity of inward K^+^ channels and stomatal aperture. Furthermore, we speculate that some signaling pathways may also participate in the regulation of KAT channels in rice guard cells by modulating the activity of this pair of channels. The findings reported here and further work hereon may provide useful information for engineering the channels or pathways and producing drought tolerant crops.

## Supporting Information

Table S1 **Primer sequences used for cloning, real-time PCR** and **RT-PCR of rice KATs.**
(DOCX)
