# Supplementary material for: Unique Features of Two Potassium Channels, OsKAT2 and OsKAT3, Expressed in Rice Guard Cells
Source: PLoS One. 2013 Aug 13;8(8):e72541. doi: 10.1371/journal.pone.0072541 (PMC3742606; doi:10.1371/journal.pone.0072541)
Supplement: Table S1 — Primer sequences used for cloning, real-time PCR and RT-PCR of rice KATs. (DOCX) [file pone.0072541.s001.docx]

Supplementary Table 1. Primer sequences used for cloning, real-time PCR and RT-PCR of rice KATs.

| **Usage** | **Name** | **Sequence (5'->3')** |
| --- | --- | --- |
| CDS cloning | *OsKAT1* | Forward ATGCCACGTTCTTCTCGTATG |
|  |  | Reverse TACGTTCACTTGCTGAAGGTTGCT |
|  | *OsKAT2* | Forward ATGGAAACTATTAGCAATATC |
|  |  | Reverse AATTTCCATAAAAAACAGATGATC |
|  | *OsKAT3* | Forward ATGACCCAAGCTCACTCAAAA |
|  |  | Reverse CATCTCAAGAAGGAATAGATGGTC |
| Promotor cloning | *OsKAT1* | Forward AGGAAGCAATGGATGTGATGGG |
|  |  | Reverse AATATTTCGGTCCAGGGCGACG |
|  | *OsKAT2* | Forward AGGCTTAATAAATTTGTCTTGTGGT |
|  |  | Reverse GTTAGAAGTGGGAGGTGCCTAAGT |
|  | *OsKAT3* | Forward CAGTGCTGAACAAGGCCCTTCAAA |
|  |  | Reverse TTGCTGTTCTTGTTCTTGGCCACC |
| Real time and | *UBi5* | Forward ACCACTTCGACCGCCACTACT |
| RT-PCR |  | Reverse ACGCCTAAGCCTGCTGGTT |
|  | *OsKAT1* | Forward ACATATGCCTCCGGTACA |
|  |  | Reverse GAGACTCCCTTGAACAGGTA |
|  | *OsKAT2* | Forward CGAGTTGCAGTCCTGTAGTT |
|  |  | Reverse TCCACAGATCATAGAGGGC |
|  | *OsKAT3* | Forward CCAGACCGAAGATTCTTGAG |
|  |  | Reverse CTGTCACCTCCAAATACTGG |
